# Falls in older aged adults in 22 European countries: incidence, mortality and burden of disease from 1990 to 2017

**DOI:** 10.1136/injuryprev-2019-043347

**Published:** 2020-02-28

**Authors:** Juanita A Haagsma, Branko F Olij, Marek Majdan, Ed F van Beeck, Theo Vos, Chris D Castle, Zachary V Dingels, Jack T Fox, Erin B Hamilton, Zichen Liu, Nicholas L S Roberts, Dillon O Sylte, Olatunde Aremu, Till Winfried Bärnighausen, Antonio M Borzì, Andrew M Briggs, Juan J Carrero, Cyrus Cooper, Ziad El-Khatib, Christian Lycke Ellingsen, Seyed-Mohammad Fereshtehnejad, Irina Filip, Florian Fischer, Josep Maria Haro, Jost B Jonas, Aliasghar A Kiadaliri, Ai Koyanagi, Raimundas Lunevicius, Tuomo J Meretoja, Shafiu Mohammed, Ashish Pathak, Amir Radfar, Salman Rawaf, David Laith Rawaf, Lidia Sanchez Riera, Ivy Shiue, Tommi Juhani Vasankari, Spencer L James, Suzanne Polinder

**Affiliations:** 1 Department of Public Health, Erasmus University Medical Center, Rotterdam, The Netherlands; 2 Consumer Safety Institute, Amsterdam, The Netherlands; 3 Department of Public Health, Trnava University, Trnava, Slovakia; 4 Department of Public Health, Erasmus University, Rotterdam, The Netherlands; 5 Institute for Health Metrics and Evaluation, University of Washington, Seattle, Washington, USA; 6 Department of Health Metrics Sciences, School of Medicine, University of Washington, Seattle, WA, USA; 7 School of Health Sciences, Birmingham City University, Birmingham, UK; 8 Heidelberg Institute of Global Health (HIGH), Heidelberg University, Heidelberg, Germany; 9 T.H. Chan School of Public Health, Harvard University, Boston, Massachusetts, USA; 10 General Surgery and Medical-Surgical Specialties, University of Catania, Catania, Italy; 11 School of Physiotherapy and Exercise Science, Curtin University, Bentley, Western Australia, Australia; 12 Ageing and Life Course, World Health Organization (WHO), Geneva, Switzerland; 13 Department of Medical Epidemiology and Biostatistics, Karolinska Institutet, Stockholm, Sweden; 14 Medical Research Council Lifecourse Epidemiology Unit, University of Southampton, Southampton, UK; 15 Department of Rheumatology, University of Oxford, Oxford, UK; 16 Department of Public Health Sciences, Karolinska Institutet, Stockholm, Sweden; 17 Department of Pathology, Stavanger University Hospital, Stavanger, Norway; 18 Norwegian Institute of Public Health, Oslo, Norway; 19 Department of Neurobiology, Care Sciences and Society, Karolinska Institutet, Stockholm, Sweden; 20 Division of Neurology, University of Ottawa, Ottawa, Ontario, Canada; 21 Psychiatry Department, Kaiser Permanente, Fontana, California, USA; 22 School of Health Sciences, A.T. Still University, Mesa, Arizona, USA; 23 School of Public Health Medicine, Bielefeld University, Bielefeld, Germany; 24 Biomedical Research Networking Center for Mental Health Network (CiberSAM), Madrid, Spain; 25 Research and Development Unit, San Juan de Dios Sanitary Park, Sant Boi de Llobregat, Spain; 26 Department of Ophthalmology, Heidelberg University, Mannheim, Germany; 27 Beijing Institute of Ophthalmology, Beijing Tongren Hospital, Beijing, China; 28 Clinical Epidemiology Unit, Lund University, Lund, Sweden; 29 CIBERSAM, San Juan de Dios Sanitary Park, Sant Boi de Llobregat, Spain; 30 Catalan Institution for Research and Advanced Studies (ICREA), Barcelona, Spain; 31 Department of General Surgery, Aintree University Hospital National Health Service (NHS) Foundation Trust, Liverpool, UK; 32 Department of Surgery, University of Liverpool, Liverpool, UK; 33 Breast Surgery Unit, Helsinki University Hospital, Helsinki, Finland; 34 University of Helsinki, Helsinki, Finland; 35 Health Systems and Policy Research Unit, Ahmadu Bello University, Zaria, Nigeria; 36 Institute of Public Health, Heidelberg University, Heidelberg, Germany; 37 Department of Pediatircs, RD Gardi Medical College, Ujjain, India; 38 College of Graduate Health Sciences, A.T. Still University, Mesa, Arizona, USA; 39 Medichem, Barcelona, Spain; 40 Department of Primary Care and Public Health, Imperial College London, London, UK; 41 Academic Public Health, Public Health England, London, UK; 42 WHO Collaborating Centre for Public Health Education and Training, Imperial College London, London, UK; 43 University College London Hospitals, London, UK; 44 Department of Rheumatology, University Hospitals Bristol NHS Foundation Trust, Bristol, UK; 45 Institute of Bone and Joint Research, University of Sydney, Sydney, New South Wales, Australia; 46 Institute of Medical Epidemiology, Martin Luther University Halle-Wittenberg, Halle, Germany; 47 UKK Institute, Tampere, Finland

**Keywords:** disability, burden of disease, time series, metanalysis

## Abstract

**Introduction:**

Falls in older aged adults are an important public health problem. Insight into differences in fall-related injury rates between countries can serve as important input for identifying and evaluating prevention strategies. The objectives of this study were to compare Global Burden of Disease (GBD) 2017 estimates on incidence, mortality and disability-adjusted life years (DALYs) due to fall-related injury in older adults across 22 countries in the Western European region and to examine changes over a 28-year period.

**Methods:**

We performed a secondary database descriptive study using the GBD 2017 results on age-standardised fall-related injury in older adults aged 70 years and older in 22 countries from 1990 to 2017.

**Results:**

In 2017, in the Western European region, 13 840 per 100 000 (uncertainty interval (UI) 11 837–16 113) older adults sought medical treatment for fall-related injury, ranging from 7594 per 100 000 (UI 6326–9032) in Greece to 19 796 per 100 000 (UI 15 536–24 233) in Norway. Since 1990, fall-related injury DALY rates showed little change for the whole region, but patterns varied widely between countries. Some countries (eg, Belgium and Netherlands) have lost their favourable positions due to an increasing fall-related injury burden of disease since 1990.

**Conclusions:**

From 1990 to 2017, there was considerable variation in fall-related injury incidence, mortality, DALY rates and its composites in the 22 countries in the Western European region. It may be useful to assess which fall prevention measures have been taken in countries that showed continuous low or decreasing incidence, death and DALY rates despite ageing of the population.

## Introduction

Falls are common and may lead to a large deterioration in health among older adults. The Western European region is one of the world regions with the highest fall-related injury incidence and mortality rates in older aged adults.^1^

Insight into differences in fall-related injury rates between countries can serve as important input for identifying effective prevention strategies. However, intercountry comparisons are hampered because often different methodologies are used to assess fall-related injury rates.^2–5^ Studies that did use a similar methodology focused either on fall-related injury incidence or mortality.^6–9^ A major shortcoming of this is that injuries resulting from falls show great variety in severity and duration, and, consequently, using incidence or mortality rates only partially gives an indication of the population health impact of falls.

A measure that includes mortality and morbidity is the disability-adjusted life year (DALY). The DALY is a composite measure that aggregates premature mortality and disability into a single metric, thus providing a more comprehensive measure of the relative health impact of public health problems compared with mortality or incidence figures alone.^10^

A landmark study that used the DALY is the Global Burden of Disease and Injury study. The Global Burden of Disease (GBD) study annually quantifies mortality, incidence, prevalence and DALYs for over 300 diseases and causes of injury of 195 countries and territories using a standardised and systematic approach.^11–16^ This strategy results in internally consistent and comparable estimates, both between populations and over time.

The objectives of this study were to

1. Compare GBD 2017 estimates on fall-related injury incidence, mortality and DALYs and its components in older adults across 22 countries of the Western European region.
2. Examine changes of DALYs due to fall-related injury for these 22 countries over a 28-year period.

## Methods

We analysed levels and trends of incidence, mortality and DALY and its components, years of life lost (YLLs) and years lived with disability (YLDs) of falls injury in adults aged 70 years and older in the Western European region of the GBD 2017 study.^16^ The DALY is calculated by adding YLLs and YLDs. YLLs are calculated by multiplying deaths by the remaining life expectancy at the age of death. YLDs are calculated by multiplying the number of cases with a certain health outcome and by the disability weight assigned to this health outcome.

The overall GBD 2017 study provided global and regional estimates for 359 diseases and injuries for 23 age groups, both sexes, and 195 countries and territories from 1990 to 2017.^16^ Detailed descriptions of the methodology and approach of the GBD study and supplemental information on methods that were used to calculate incidence, mortality, YLL, YLD and DALY estimates have been published elsewhere.^1 16–18^ For the present study, we used the GBD 2017 interactive data visualisation tool ‘GBD Compare’ to retrieve the estimates for falls incidence, mortality, YLLs, YLDs and DALYs of older adults (GBD 2017 Results. Seattle, United States: Institute for Health Metrics and Evaluation (IHME), 2017; http://vizhub.healthdata.org/gbd-compare/). In our study, older adults are defined as individuals who are 70 years and older. We used estimates for each year in the period of 1990–2017. We compared both total numbers and rates of falls incidence, mortality, YLD, YLL and DALY by age category (70–74, 75–80, 80–84, 85–90, 90–94 and 95+ years), sex, country and over time. The 70+-year rates by country and by year were age standardised within the 70+-year age group using direct standardisation methods.

### Countries in the Western European region

In GBD 2017, Europe is divided into three regions: the Central European region (13 countries), the Eastern European region (7 countries) and the Western European region (22 countries). The following countries were included in the Western European region of the GBD: Andorra, Austria, Belgium, Cyprus, Denmark, Finland, France, Germany, Greece, Iceland, Ireland, Israel, Italy, Luxembourg, Malta, the Netherlands, Norway, Portugal, Spain, Sweden, Switzerland and the UK.

### GBD falls injury classification

Injury incidence and mortality data coded according to the International Classification of Diseases, Ninth Revision (ICD-9) and the International Statistical Classification of Diseases and Related Health Problems, 10th Revision (ICD-10) were categorised into mutually exclusive and collectively exhaustive GBD nature-of-injury categories.^1 16^ The detailed list of ICD-9 and ICD-10 codes was provided elsewhere.^1 16–18^ Fall-related injury incidence and death were defined as in ICD-9 codes E880-888 and E929.3 and ICD-10 codes W100-W19. Morbidity analysis was restricted to cases warranting some form of healthcare in a healthcare system where patients have full, unrestricted access to healthcare. This includes (1) injury cases of sufficient severity to require inpatient care and (2) injury cases of sufficient severity to require healthcare attention but not hospitalisation.^1^ This latter category includes emergency department and general practitioner visits.

### Uncertainty

The GBD estimates have varying degrees of uncertainty in the input data, the data adjustments and the statistical models used to estimate values for all countries over time. Standard GBD methodology is that for each component (incidence, mortality, YLD, YLL and DALY), uncertainty from each source is propagated at the level of 1000 draws; that is, all estimates were calculated 1000 times, each time drawing from distributions. In the Results section, we present the median value of the 1000 draws of the sampled incidence, mortality, YLD, YLL and DALY values. We also present the uncertainty interval (UI), which corresponds to the 2.5th and 97.5th percentiles of the range of values resulting from the 1000 draws of the corresponding distribution.

## Results

### Mortality and incidence

In 2017, in the Western European region, 11.7 million (UI 10.3–13.2 million) adults aged 70 and older sought medical attention due to an injury, of which 8.4 million (71.9%, UI 7.2–9.8 million) were due to fall-related injury. In 2017, 54 504 (UI 52 385–56 650) older adults died due to falls. The incidence rate of fall-related injury requiring healthcare increased substantially by age, with an incidence rate of 5667 (UI 3999–7625) per 100 000 in the age category of 70–74 years to 47 239 (UI 33 684–63 127) per 100 000 in the age category of 95+ years. For death due to a fall, this increase was even more pronounced, with death rates ranging from 18 (UI 17–19) per 100 000 in the age category of 70–74 years to 705 (UI 666–748) per 100 000 in the age category of 95+ years. The incidence rate of fall-related injury requiring healthcare was higher in women than in men (women: 16 958 (UI 14 487–19 772) vs men: 9596 (UI 8127–11 311)); however, death rates in older adults were slightly, but not significantly, higher in men (women: 89 (UI 84–94) vs men: 91 (UI 86–96)).

Incidence rates of fall-related injury requiring healthcare in older adults varied widely by country, with the lowest incidence rates in Greece (7594 per 100 000 (UI 6326–9032)) and Portugal (8086 per 100 000 (UI 6790–9659)), and the highest incidence rates in Belgium (19 634 per 100 000 (UI 16 497–23 644)) and Norway (19 796 per 100 000 (UI 15 536–24 233)). Death rates were also lowest in Greece (29 per 100 000 (UI 27–31)) and Portugal (36 per 100 000 (UI 33–39)), and highest in Norway (153 per 100 000 (UI 147–159)) and Switzerland (153 per 100 000 (UI 141–166)). The case fatality rate (the death rate/incidence rate) was highest in the Netherlands (1.1%) and Switzerland (0.8%), twice as high compared with the countries with the lowest case fatality rates (Portugal (0.4%) and Greece (0.4%)). Table 1 shows the incidence and death rates of falls in older adults by country.

**Table 1:** Incidence and death rates of falls in older adults (70+ years) per 100 000 by country with 95% uncertainty intervals, 2017

Country	Incidence rate* (per 100 000)	Rank number incidence rate†	Death rate* (per 100 000)	Rank number death rate†	Total deaths (%)‡	Case fatality rate§
Andorra	15 556 (12 964–18 709)	7	88.6 (71.8–107.8)	12	1.7	0.006
Austria	14 863 (12 617–17 445)	9	96.8 (89.0–105.1)	10	1.8	0.007
Belgium	19 634 (16 498–23 644)	2	118.4 (108.3–128.9)	6	2.1	0.006
Cyprus	9964 (8260–12 017)	19	54.9 (47.3–62.8)	17	1.2	0.006
Denmark	13 620 (11 496–16 188)	13	97.2 (89.7–106.1)	9	1.8	0.007
Finland	18 808 (15 864–22 068)	4	132.5 (123.2–142.6)	5	2.5	0.007
France	17 682 (14 941–20 963)	6	133.5 (122.1–145.4)	4	2.7	0.008
Germany	14 962 (12 556–17 604)	8	95.3 (85.8–105.9)	11	1.6	0.006
Greece	7594 (6326–9032)	22	29.0 (26.7–31.5)	22	0.5	0.004
Iceland	13 312 (11 266–15 555)	14	87.6 (80.8–95.0)	13	1.7	0.007
Ireland	10 489 (8826–12 502)	17	54.2 (49.6–59.6)	18	1.1	0.005
Israel	8811 (7438–10 453)	20	44.4 (40.5–48.7)	20	0.9	0.005
Italy	12 850 (10 899–15 215)	15	69.0 (63.3–75.2)	16	1.3	0.005
Luxembourg	17 713 (14 791–21 045)	5	113.6 (101.1–127.7)	7	2.0	0.006
Malta	13 654 (11 630–16 059)	11	77.2 (70.8–85.0)	15	1.5	0.006
Netherlands	13 623 (11 756–15 894)	12	145.5 (133.8–157.8)	3	2.7	0.011
Norway	19 796 (15 536–24 233)	1	152.6 (146.6–158.8)	2	2.8%	0.008
Portugal	8086 (6790–9659)	21	35.9 (32.8–38.9)	21	0.6	0.004
Spain	10 161 (8571-–12 003)	18	50.1 (46.1–54.6)	19	1.0	0.005
Sweden	14 835 (11 751–18 249)	10	103.1 (95.8–110.5)	8	2.0%	0.007
Switzerland	19 431 (17 099–22 400)	3	153.2 (141.3–165.9)	1	3.3	0.008
UK	12 099 (9814–14 585)	16	78.6 (77.0–80.4)	14	1.4	0.006

*The 70+ incidence and mortality rates by country were age standardised within the 70+-year age group.

†Rank numbers based on values from highest (1) to lowest (22).

‡Per cent of total deaths is the relative contribution of falls deaths to the total deaths of all causes in the population aged 70 years and older.

§Case fatality rate=death rate/incidence rate.

### Burden of disease

In 2017, the total burden of disease due to injuries in older adults in the Western European region was 2.5 million DALYs (UI 2.0–3.0 million), of which 1.4 million DALYs (54.5%, UI 1.1–1.7 million) were due to fall-related injury. YLLs were responsible for 33.5% of falls DALYs (453 213 YLLs (UI 433 949–471 961)) and YLDs for 66.5% of fall-related injury DALYs (897 968 YLDs (UI 632 890–1 221 547)). The DALY, YLL and YLD rates increased with age.

**Figure 1:**
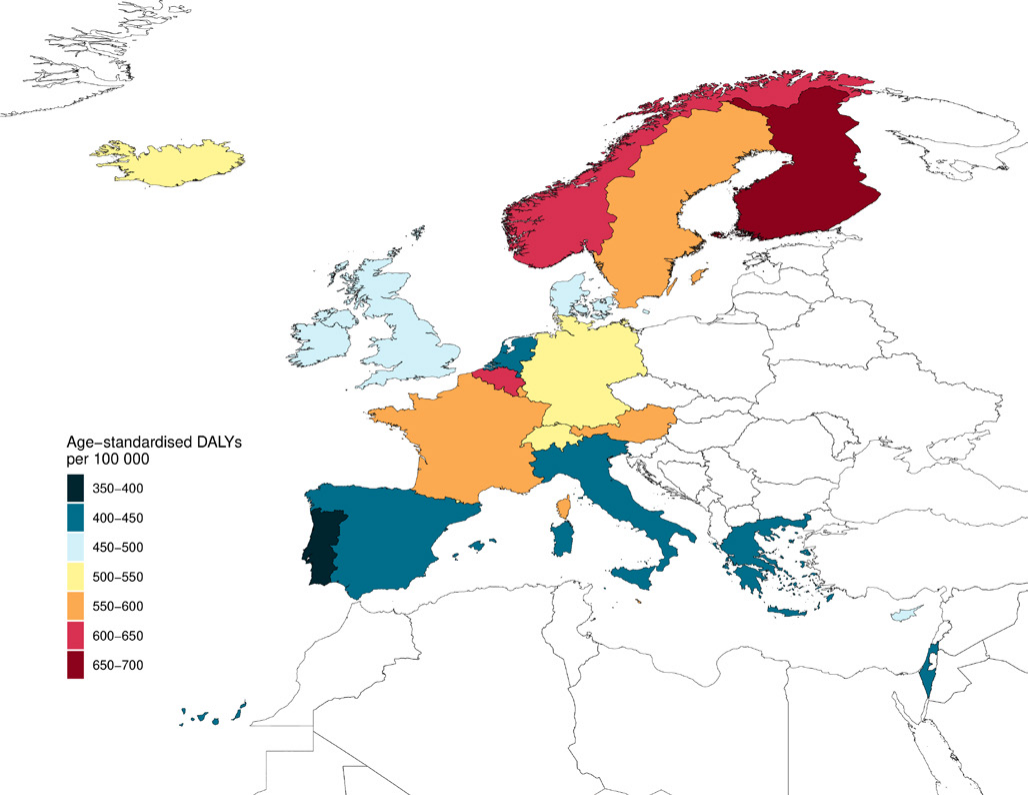
Age-standardised DALY rate (the 70+ DALY rates by country were age standardised within the 70+-year age group) of falls in older adults per 100 000 per country, 2017. DALY, disability-adjusted life year.

Figure 1 shows the age standardised DALY rates per country. Table 2 shows the DALY, YLL and YLD rates per country. DALY rates of falls in older adults were lowest in Portugal (1335 DALYs per 100 000 (UI 1042–1694) and Greece (1356 DALYs per 100 000 (UI 1025–1757)) and highest in Norway (3126 DALYs per 100 000 (UI 2555–3796)) and Finland (3133 per 100 000 (UI 2533–3812). The relative contribution of fall-related injury DALYs to the total DALYs due to all causes in the population aged 70 years and older was highest in Norway (4.1%), Finland (4.1%), France (4.1%) and Switzerland (4.5%).

**Table 2:** DALY, YLD and YLL rates of falls in older adults (70+) per 100 000 by country with 95% uncertainty intervals, 2017

Country	YLD rate* (per 100 000)	YLL rate* (per 100 00)	DALY rate* (per 100 00)	Rank number DALY rate†	Total DALYs (%)‡
Andorra	1654 (1167–2237)	710 (575–875)	2363 (1843–2921)	11	3.2
Austria	1585 (1114–2173)	866 (793–946)	2451 (1971–3038)	9	3.2
Belgium	2017 (1416–2746)	1006 (918–1095)	3024 (2431–3744)	4	3.7
Cyprus	1219 (852–1676)	524 (451–603)	1744 (1359–2202)	18	2.3
Denmark	1381 (983–1889)	782 (715-853)	2162 (1764–2655)	14	2.7
Finland	1945 (1365–2637)	1189 (1097–1284)	3133 (2533–3812)	1	4.1
France	1806 (1274–2452)	1006 (915–1098)	2812 (2271–3449)	5	4.1
Germany	1536 (1073–2091)	850 (761–950)	2386 (1939–2928)	10	2.9
Greece	1079 (758–1485)	277 (255-302)	1356 (1025–1757)	21	1.7
Iceland	1457 (1030–1989)	722 (663–785)	2179 (1732–2707)	13	2.9
Ireland	1260 (892–1721)	492 (445–544)	1752 (1375–2210)	17	2.3
Israel	1142 (807–1563)	360 (327–395)	1503 (1166–1912)	20	2.1
Italy	1356 (952–1851)	561 (518–615)	1917 (1514–2392)	16	2.6
Luxembourg	1709 (1206–2326)	972 (853–1096)	2681 (2171–3277)	6	3.3
Malta	1511 (1065–2067)	723 (658–794)	2234 (1762–2782)	12	2.8
Netherlands	1299 (918–1761)	1188 (1097–1282)	2487 (2094–2966)	8	3.1
Norway	1944 (1369–2608)	1182 (1133–1236)	3126 (2555–3796)	2	4.1
Portugal	997 (698–1363)	339 (306–369)	1335 (1042–1694)	22	1.7
Spain	1246 (878–1701)	429 (393–467)	1675 (1303–2127)	19	2.3
Sweden	1672 (1178–2235)	833 (775–897)	2505 (2004–3071)	7	3.4
Switzerland	1884 (1331–2535)	1198 (1101–1302)	3082 (2508–3744)	3	4.5
UK	1369 (966–1861)	671 (656–688)	2041 (1633–2527)	15	2.5

*The 70+YLD, YLL and DALY rates by country were age standardised within the 70+-year age group.

†Rank numbers based on values from highest (1) to lowest (22).

‡Per cent of total DALYs is the relative contribution of falls DALYs to the total DALYs of all causes in the population aged 70 years and older.

DALY, disability-adjusted life year; YLD, year lived with disability; YLL, year of life lost.

### Changes in burden of disease due to fall-related injury, 1990–2017

**Figure 2:**
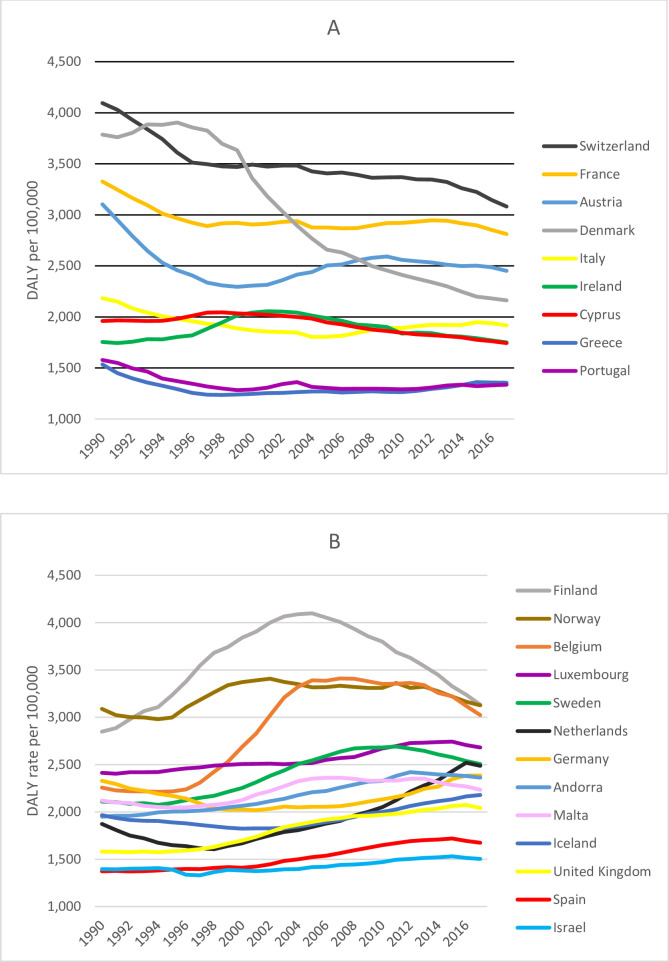
DALY rate (the 70+ DALY rates by country were age standardised within the 70+-year age group) of falls in older adults per 100 000 per country in the period from 1990 to 2017. (A) Countries with a decrease in DALY rate between 1990 and 2017. (B) Countries with an increase in DALY rate between 1990 and 2017. DALY, disability-adjusted life year.

The number of DALYs due to fall-related injury in older adults increased by 54%, from 837 679 DALYs (UI 693 158–1 023 106) in 1990 to 1 351 181 DALYs (UI 1 086 838–1 667 340) in 2017. However, the rate of DALYs due to fall-related injury showed little change over time from 2245 DALYs per 100 000 (UI 1857–2741) in 1990 to 2227 DALYs per 100 000 (UI 1791–2568) in 2017. Trends in DALY rates of fall-related injury in older adults over the period from 1990 to 2017 varied widely, from large decreases in Denmark (−42.9%), Switzerland (−24.7%) and Austria (−21.0%) to large increases in the UK (29.0%), the Netherlands (32.8%) and Belgium (34.0%). This resulted in countries losing their favourable positions in terms of fall-related injury DALYs compared with other countries in the Western European region. Finland stands out because DALY rates of fall-related injury in older adults rapidly increased from 1990 to 2005, followed by a decline. A similar, but less pronounced, pattern was seen in Belgium. Denmark also stands out because fall-related DALY rates slightly increased between 1990 and 1997, followed by a rapid decline between 1999 and 2017. Figure 2 shows the DALY rate per country from 1990 to 2017. Table 3 shows the 1990 fall-related injury DALY rates and per cent of change.

**Table 3:** DALY rates and per cent of change* of falls in the elderly (70+ years) per 100 000 by country, 1990–2017

Country	DALY rate† per 100 000 in 1990	Rank number DALY rate 1990‡	Per cent of change (%)* (1990–2017)
Andorra	1950 (1511–2441)	15	21.2
Austria	3103 (2601–3734)	4	−21.0
Belgium	2257 (1824–2807)	9	34.0
Cyprus	1959 (1554–2425)	14	−11.0
Denmark	3785 (3260–4438)	2	−42.9
Finland	2848 (2364–3456)	6	10.0
France	3326 (2774–4006)	3	−15.5
Germany	2328 (1910–2848)	8	2.5
Greece	1535 (1205–1937)	20	−11.7
Iceland	1965 (1584–2434)	13	10.9
Ireland	1755 (1424–2170)	17	−0.2
Israel	1396 (1123–1738)	21	7.6
Italy	2183 (1800–2668)	10	−12.2
Luxembourg	2412 (1961–2960)	7	11.1
Malta	2119 (1730–2568)	11	5.4
Netherlands	1872 (1537–2274)	16	32.8
Norway	3088 (2566–3710)	5	1.2
Portugal	1578 (1275–1958)	19	−15.4
Spain	1372 (1068–1749)	22	22.1
Sweden	2110 (1724–2584)	12	18.7
Switzerland	4095 (3451–4853)	1	−24.7
UK	1582 (1260–1979)	18	29.0

*The percent of change is the percentage *change* in DALY rate in the period from 1990 to 2017. A positive percentage of change indicates an increase; a negative percentage of change indicates a decrease.

†The 70+ DALY rates by country were age standardised within the 70+age group.

‡Rank numbers based on values from highest 1 to lowest 22

### Changes in YLD and YLL, 1990–2017

**Figure 3:**
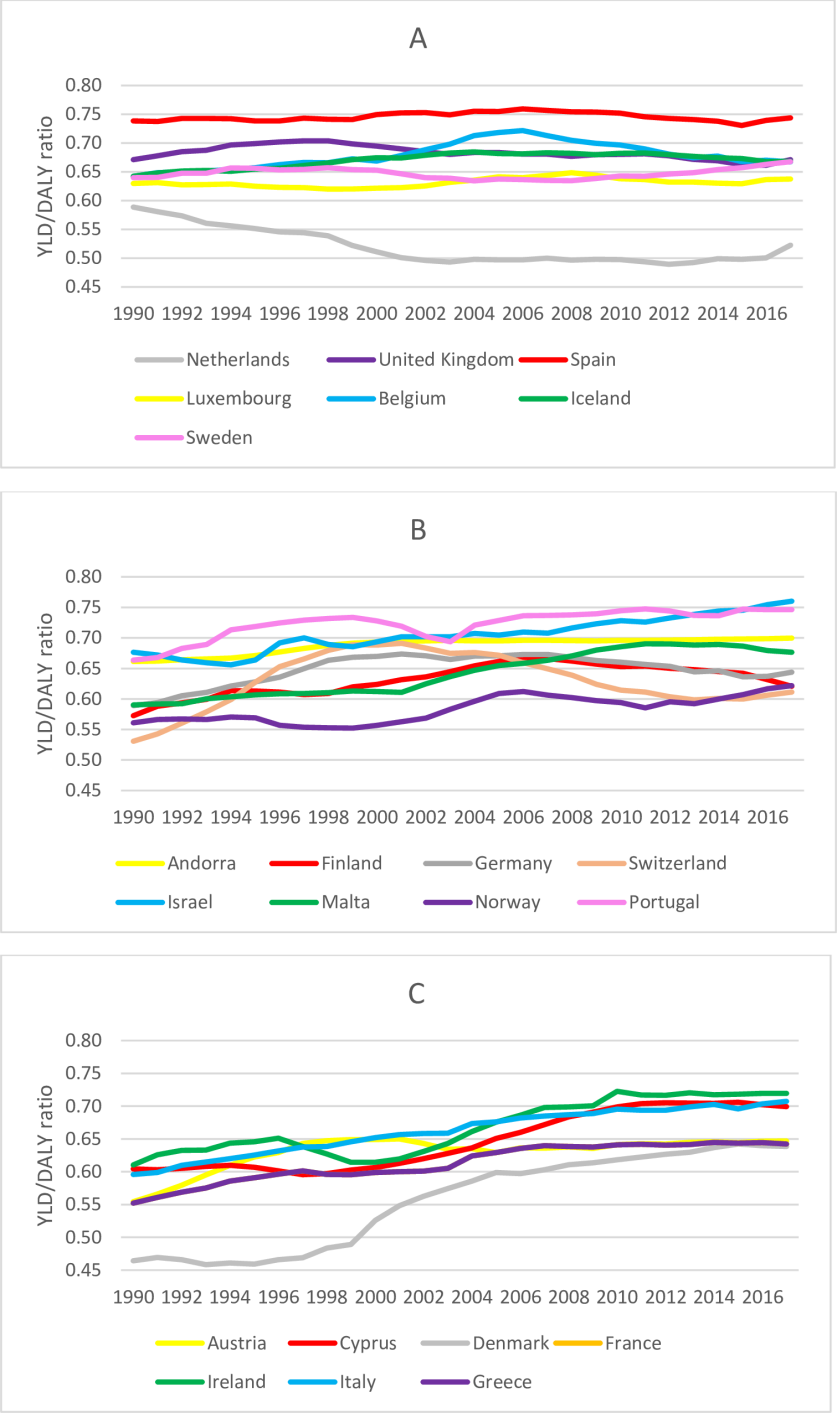
YLD:DALY ratio of falls in older adults by country, 1990–2017. (A) Countries with a decrease or a small increase (<0.04) in YLD:DALY ratio between 1990 and 2017. (B) Countries with a medium increase (≥0.04 and<0.09) in YLD:DALY ratio between 1990 and 2017. (C) Countries with a large increase (≥0.09) in YLD:DALY ratio between 1990 and 2017. DALY, disability-adjusted life year; YLD, year lived with disability.

Between 1990 and 2017, falls YLL rates declined significantly by 16.7%, respectively, whereas fall-related injury YLD rates showed a slight increase (not significantly increased by 9.8%), indicating a shift towards YLD as the primary driver of fall-related injury DALYs in older adults. This shift was apparent for most countries but not at the same rate. Largest increases in YLD:DALY ratio were found in Ireland (1990: 61%, 2017: 72%), Italy (1990: 60%, 2017: 71%) and Denmark (1990: 46%, 2017: 64%). Smallest increases in YLD:DALY ratio were found in the UK (1990: 67%, 2017: 67%), Spain (1990: 74%, 2017: 74%) and Luxembourg (1990: 63%, 2017: 64%). The Netherlands was the only country that showed a decrease in the YLD:DALY ratio between 1990 and 2017 (1990: 59%, 2017: 52%). Figure 3 shows the YLD:DALY ratio for the 22 countries over the period of 1990–2017.

## Discussion

Incidence, mortality and DALY rates of fall-related injury in older adults varied widely by Western European country. There was a fivefold difference in death rates due to fall-related injury between the countries with lowest and highest falls death rates. For incidence and DALY rates, the difference between countries with the highest and lowest rates was twofold.

The fall-related death and injury incidence rates in older adults from the GBD 2017 study are higher compared with those reported by previously published studies.^2 3 8 19–22^ These differences in incidence and mortality rates may be explained by broader age ranges included in the previously published studies. Typically, incidence and mortality rates of falls in older adults increase with age, and we have restricted our study to the age category of 70 years and older rather than 60 or 65 years and older, which may have led to higher incidence and mortality rates.

A second explanation for the difference in incidence rates may be that a different case definition was applied. Often, studies reported incidence rates of cases admitted to hospital, whereas the GBD analysis covers cases warranting some form of healthcare in a system. This includes patients who visited the emergency department due to fall-related injuries. A Belgian study that assessed the incidence of fall-related injury in older adults and that included primary care visits and emergency department-reported falls injury incidence rates similar to the GBD.^23^

Third, the GBD corrects for ill-defined and unknown causes of death in cause-of-death registries.^24^ Ill-defined deaths can be subdivided into two categories: general ill-defined and unknown cause of death (eg, R99 Ill-defined and unknown cause of mortality) and injury ill-defined cause of death (eg, X59 Exposure to unspecified factor). Both types of ill-defined and unknown causes of death were proportionally redistributed on all injury codes, including falls.^24^ For the specific nature of injury codes such as falls redistribution of general ill-defined and unknown deaths leads to a small number of redistributed deaths and subsequently a small increase in death rates. The second category of ill-defined and unknown deaths will be redistributed within injury causes only; hence, redistribution of this category of ill-defined and unknown deaths will proportionally lead to a higher increase in fall death rates. The total increase of fall death rates (and other nature of injury categories) depends on the total percentage of ill-defined and unknown deaths in cause of death registries, and this percentage varies by country and by year.

An important finding of this study is that since 1990, DALY rates due to fall-related injury showed little change for the whole region, but patterns varied widely between countries. In Denmark, Switzerland and Austria, the burden of fall-related injury in older adults decreased substantially, whereas other countries (eg, the Netherlands and Belgium) have lost their favourable positions in terms of fall-related injury DALYs due to an increasing fall-related burden of disease since 1990. Researchers have identified several main risk factors for falls in older adults, and the combination of each of these risk factors may vary by country and over time, making it difficult to unravel which prevention measures have yielded the largest effect.^6 25^ Nevertheless, it may be useful to assess which fall prevention measures have been taken in countries that showed continuous low or decreasing incidence, death and DALY rates despite ageing of the population. In addition to fall prevention measures, there may be important differences in the delivery of care following a fall-related injury that may affect injury outcome. If rates of fall-related injury in the elderly can be lowered to those of countries with the lowest levels in 2017, potentially 892 DALYs per 100 000 could be averted in the Western European region.

A second important finding is that the YLL rates decreased significantly, whereas YLD rates showed little change over time, indicating a shift towards YLD as the primary driver of fall-related injury DALYs in older adults. The rate of this shift varied tremendously between countries. The shift towards YLD may be the result of improved access to better quality care after sustaining an injury or by fall prevention measures that resulted in a reduction of the severity of injury sustained due to a fall. Another explanation may be that frailty, a major risk factor of falls in older adults, and chronic disease and disabilities occur at higher ages compared with 1990, resulting in a shift off falls incidence and mortality towards the very old ages.^12 25^

### Strengths and limitations

A major strength of our study is the internal consistency and comparability of the incidence, mortality, YLD, YLL and DALY estimates, which allow comparison of these results over time and across countries. A second strength is that the death rate estimates in Western European countries were based on complete vital registration systems. However, nationally representative incidence data on fall-related injury were available for five countries only (Belgium, Finland, the Netherlands, Portugal and Switzerland). Incidence estimates for every Western European country were made by using statistical models that use available data on incidence, prevalence, remission, duration and extra risk of mortality due to the injury from the year and country for which incidence is estimated, as well as from previous years and other countries, but these estimates are inherently less precise for countries without national representative incidence data.^26^

The European Hospital Morbidity Database was an important data source for the five countries for which nationally representative injury incidence data were available. However, these data were available only in tabular form, and oftentimes, the European Hospital Morbidity Database registered the nature of injury categories as the underlying cause of injury, making it impossible to derive incidence by the actual cause of injury (eg, falls). The GBD estimates for injuries would be greatly strengthened if hospital data were made available in microdata form and with multiple diagnosis fields.

The Netherlands was the only country that provided emergency department data on injuries, but this information is most probably available for many Western European countries as well. Availability of cause and nature of injury coded emergency department data for other countries will also improve the GBD injury estimates greatly.

Another limitation of this study is that the DALY estimates were based on prevalence-based data. DisMod-MR is used to estimate prevalence from incidence, and this process assumes a steady state where rates are not changing over time. This steady-state assumption may lead to inaccurate estimates of prevalence of long-term disability if there are large trends in incidence rates or mortality.

Lastly, in our study, all fall-related injuries were classified as falls, whereas a more detailed classification of falls (eg, single-level falls vs falls from height) and information on other circumstances regarding the fall (eg, location of the fall) may help to explain differences in patterns over time and across countries and may provide important input for fall prevention.

## Conclusions and implications for policy

In conclusion, there is considerable variation in incidence, mortality and DALY rates of fall-related injury in older adults in the 22 countries of the Western European region. Since 1990, the burden of disease of fall-related injury showed little change in the whole region, but patterns vary between countries. It may be useful to assess which fall prevention, healthcare and rehabilitation measures have been taken in countries that showed continuous low or decreasing incidence, death and DALY rates despite ageing of the population. Furthermore, estimates for fall-related injury incidence, mortality and DALY would be greatly strengthened if more detailed cause and nature of injury hospital and emergency department data were made available.

**What is already known on the subject**

- Falls in older aged adults are an important public health problem.
- The Western European region is one of the world regions with the highest fall-related injury incidence and mortality rates in older aged adults.
- Insight into differences in fall-related injury rates between countries can serve as important input for identifying and evaluating prevention strategies.

**What this study adds**

- From 1990 to 2017, disability adjusted life year (DALY) rates due to fall-related injury showed little change for the whole Western European region, but patterns varied widely between countries.
- Years of life lost rates decreased significantly, whereas years lived with disability (YLD) rates showed little change over time, indicating a shift towards YLD as the primary driver of fall-related injury DALYs in older adults.
- The rate of the shift towards YLD as the primary driver of fall-related injury DALYs in older adults varied tremendously between countries.
